# Prescription of Antipsychotic Medications in Delirium in Older Adults in a General Hospital Setting

**DOI:** 10.1192/bjo.2025.10570

**Published:** 2025-06-20

**Authors:** Mete Demir, Daniel Waheed, Katie French, Bushra Azam

**Affiliations:** 1 Derbyshire Healthcare NHS Foundation Trust, Chesterfield, United Kingdom; 2 Tufts University, Medford, USA.; 3 Derbyshire Healthcare NHS Foundation Trust, Derby, United Kingdom

## Abstract

**Aims:** Assess whether antipsychotics were prescribed according to MHRA (2021) and NICE (2023) guidelines.

Identify areas requiring improvement in clinical practice.

**Methods:** Retrospective case audit of older adults (65+ years) referred to Liaison Psychiatry at Chesterfield Royal Hospital for confusion/delirium between 01/08/2023–31/05/2024.

Data was extracted from patient records and analysed in Microsoft Excel. Ethical approval obtained from Derbyshire Healthcare NHS Foundation Trust (26/04/2024).

**Results:** Haloperidol was the most used antipsychotic (65.38%). Lorazepam (4 cases) or no medication (1 case) was used instead when contraindications were present.

Antipsychotic use was predominantly guided by clinical presentation, with most cases aligning with best practice recommendations. Patients who did not receive haloperidol had documented contraindications, emphasising appropriate clinical decision-making.

100% of patients who received antipsychotics had documented distress or risk to self/others.

•Baseline ECG compliance was suboptimal (47.06%), highlighting an area for improvement. Repeated ECG monitoring after dose escalation was infrequent (5.88%), indicating a gap in guideline adherence. Some documentation gaps may have contributed to apparent non-compliance.

**Conclusion:** The assessments done by Liaison Psychiatry team were mainly compliant with the standards provided by the MHRA and NICE. Most patients (65.38%) who received an antipsychotic were prescribed haloperidol as per NICE guidelines. It was clearly documented in the medical records for all patients that their presentation was a possible risk to others or themselves. Compliance to ECG requirement and recording previous ECG were weaker; though, it is important to acknowledge that in some instances, severe patient agitation made obtaining an ECG challenging. It was felt that the urgency of situation, patient’s level of agitation and distress caused to other ward patients were the most common causes that prompted Liaison team and Acute Trust staff to prescribe haloperidol without first obtaining an ECG. It is also important to consider the possibility that lower compliance might be related to lack of documentation rather than ECG not being done.

Recommendations:

Ensure ECG is performed and documented before prescribing antipsychotics.

Record reasons if ECG is not feasible due to patient agitation.

Repeat ECG after every dose increase to monitor QTc prolongation.

Liaison team nurses to receive ECG interpretation training.

Poster to remind staff to ask for and document ECG.

Create and distribute a guideline summary to the team.

Explore feasibility of including ECG specific reminder in the new core assessment/letter template (e.g. prompt).

